# Supplementary material for: Tissue Inhibitor of Matrix Metalloproteinases-1 (TIMP-1) and Pulmonary Involvement in COVID-19 Pneumonia
Source: Biomolecules. 2023 Jun 26;13(7):1040. doi: 10.3390/biom13071040 (PMC10377146; doi:10.3390/biom13071040)

Supplementary Figure 1. Gelatin zymography gels. Two gelatin zymography gels are provided as examples. MMP-9: matrix metalloproteinase-9; MMP-2: matrix metalloproteinase-2.

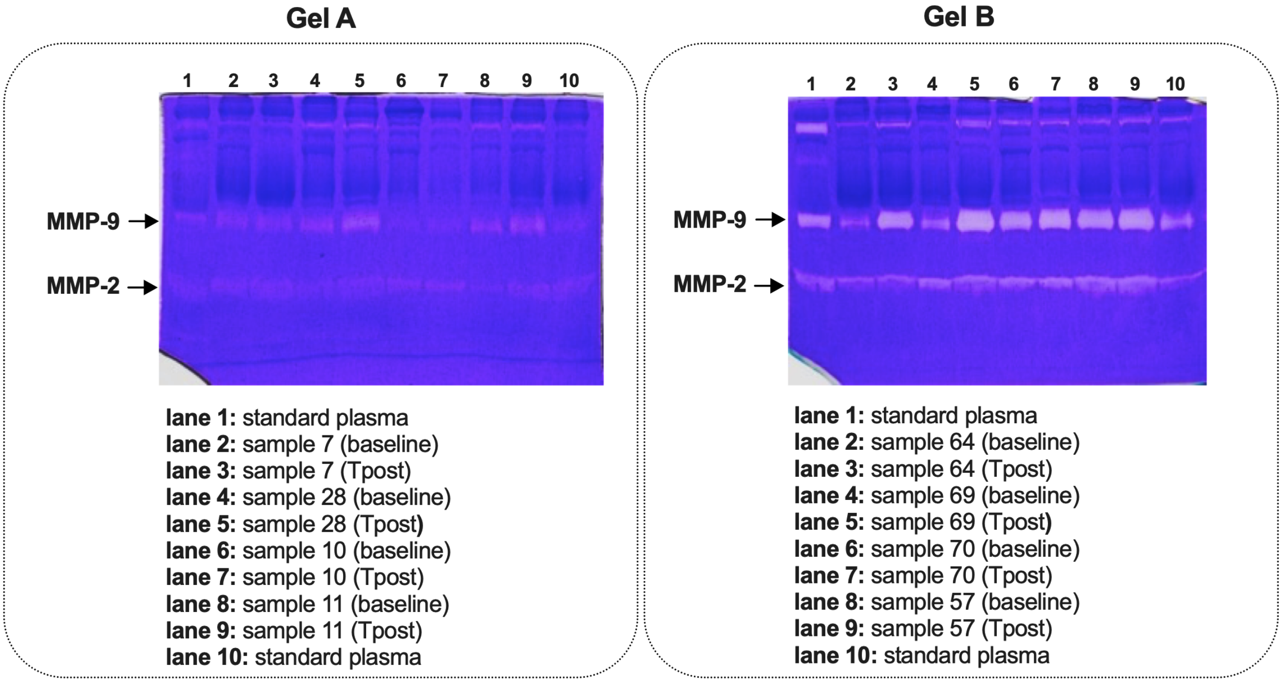

Supplement: Supplementary file 1 [file biomolecules-13-01040-s001.zip › biomolecules-2350249-supplementary.pdf]
